# Retraction: Evidence for a Pro-proliferative Feedback Loop in Prostate Cancer: The role of Epac1 and COX-2-dependent Pathways

**DOI:** 10.1371/journal.pone.0325680

**Published:** 2025-06-05

**Authors:** The PLOS One Editors

After this article [1] was published, concerns were raised regarding results presented in Figs 1, 4, 7, and 8.

Specifically:

- The following panels appear similar despite representing different experimental conditions:
  - Fig 4 p-cPLA2 1-LN and Fig 5A p-S6K^T389^ 1-LN.
  - Fig 4 p-cPLA2 PC-3 and Fig 8B COX-2 (lanes 1-6).
- The following panels presented in this article [1] appear similar to panels published in [2–10] despite representing different experimental conditions:
  - Fig 1A EP-2 PC-3 in [1] and Fig 1A IP PDK1 Raptor in [2, retracted in 3].
  - Partial or full panel similarity between Fig 1B COX-2 control for DU-145 in [1], Fig 2A Controls Raptor in [4, retracted in 5], Fig 8G S6K in [6], and Fig 5C GADD45β in [7].
  - Fig 4 COX-2 control for PC-3 in [1], Fig 2A p-TSC2 in [4, retracted in 5], Fig 4A actin in [7], Fig 4B GADPH in [7], Fig 4B FOXO1 in [7], Fig 5C in [8], and Fig 1C CREB in [9].
  - Fig 7 DU-145 control in [1], Fig 7 1-LN Actin in [1], Fig 8C Akt in [4, retracted in 5], Fig 8D (lower panel) in [4, retracted in 5], Fig 1A 1-LN actin for Bak in [10].
  - Fig 8C 1-LN Rictor in [1] and Fig 6C IB p-4EBP1 in [4, retracted in 5].

During editorial follow-up, raw blot data underlying the panels of concern were provided, however these data were insufficient to resolve the concerns listed above and raised further concerns about the labelling, storage, and reliability of the data underlying the results presented in this article.

In light of the above unresolved concerns that question the integrity and reliability of the reported results and conclusions, the *PLOS One* Editors retract this article.

SVP did not respond to the final editorial decision. UKM either could not be reached or did not respond directly.

The Fig 7 DU-145 control and I-LN Actin panels report results that appear similar to material from [10], published in 2011 by Wiley, which is not offered under a CC BY license, and are therefore excluded from this article’s [1] license.

## References

[1] Misra UK, Pizzo SV. Evidence for a pro-proliferative feedback loop in prostate cancer: the role of Epac1 and COX-2-dependent pathways. PLoS One. 2013;8(4):e63150. doi: 10.1371/journal.pone.0063150 23646189 PMC3640024

[2] Misra UK, Pizzo SV. Activated α2-macroglobulin binding to cell surface GRP78 induces T-loop phosphorylation of Akt1 by PDK1 in association with Raptor. PLoS One. 2014;9(2):e88373. doi: 10.1371/journal.pone.0088373 24516643 PMC3916429

[3] PLOS One Editors. Retraction: Activated α2-macroglobulin binding to cell surface GRP78 induces T-loop phosphorylation of Akt1 by PDK1 in association with Raptor. PLoS ONE. 2025;20(6):e325677. doi: 10.1371/journal.pone.032567740471923

[4] Misra UK, Pizzo SV. Receptor-recognized α₂-macroglobulin binds to cell surface-associated GRP78 and activates mTORC1 and mTORC2 signaling in prostate cancer cells. PLoS One. 2012;7(12):e51735. doi: 10.1371/journal.pone.0051735 23272152 PMC3522726

[5] The PLOS One Editors. Retraction: Receptor-recognized α2-macroglobulin binds to cell surface-associated GRP78 and activates mTORC1 and mTORC2 signaling in prostate cancer cells. PLoS ONE. 2025;20(6):e325675. doi: 10.1371/journal.pone.032567540471893

[6] Misra UK, Payne S, Pizzo SV. Ligation of prostate cancer cell surface GRP78 activates a proproliferative and antiapoptotic feedback loop: a role for secreted prostate-specific antigen. J Biol Chem. 2011;286(2):1248–59. doi: 10.1074/jbc.M110.129767 21056970 PMC3020732

[7] Misra UK, Deedwania R, Pizzo SV. Activation and cross-talk between Akt, NF-kappaB, and unfolded protein response signaling in 1-LN prostate cancer cells consequent to ligation of cell surface-associated GRP78. J Biol Chem. 2006;281(19):13694–707. doi: 10.1074/jbc.M511694200 16543232

[8] Misra UK, Deedwania R, Pizzo SV. Binding of activated alpha2-macroglobulin to its cell surface receptor GRP78 in 1-LN prostate cancer cells regulates PAK-2-dependent activation of LIMK. J Biol Chem. 2005;280(28):26278–86. doi: 10.1074/jbc.M414467200 15908432 PMC1201553

[9] Misra UK, Pizzo SV. Coordinate regulation of forskolin-induced cellular proliferation in macrophages by protein kinase A/cAMP-response element-binding protein (CREB) and Epac1-Rap1 signaling: effects of silencing CREB gene expression on Akt activation. J Biol Chem. 2005;280(46):38276–89. doi: 10.1074/jbc.M507332200 16172130

[10] Misra UK, Mowery YM, Gawdi G, Pizzo SV. Loss of cell surface TFII-I promotes apoptosis in prostate cancer cells stimulated with activated α₂ -macroglobulin. J Cell Biochem. 2011;112(6):1685–95. doi: 10.1002/jcb.23083 21503958 PMC3080239

